# Evidence of a cubic iron sub-lattice in t-CuFe_2_O_4_ demonstrated by X-ray Absorption Fine Structure

**DOI:** 10.1038/s41598-017-19045-8

**Published:** 2018-01-15

**Authors:** Francesco Caddeo, Danilo Loche, Maria F. Casula, Anna Corrias

**Affiliations:** 1 School of Physical Sciences, Ingram Building, University of Kent, Canterbury, CT2 7NH United Kingdom; 2 INSTM and Dipartimento di Scienze Chimiche e Geologiche, Università di Cagliari, S.S. 554, bivio per Sestu, Monserrato, CA Italy; 3 Present Address: ZIK SiLi-nano, Martin-Luther-University Halle-Wittenberg, Karl-Freiherr-von-Fritsch-Straße 3, 06120 Halle (Saale), Germany

## Abstract

Copper ferrite, belonging to the wide and technologically relevant class of spinel ferrites, was grown in the form of t-CuFe_2_O_4_ nanocrystals within a porous matrix of silica in the form of either an aerogel or a xerogel, and compared to a bulk sample. Extended X-ray absorption fine structure (EXAFS) spectroscopy revealed the presence of two different sub-lattices within the crystal structure of t-CuFe_2_O_4_, one *tetragonal* and one *cubic*, defined by the Cu^2+^ and Fe^3+^ ions respectively. Our investigation provides evidence that the Jahn-Teller distortion, which occurs on the Cu^2+^ ions located in octahedral sites, does not affect the coordination geometry of the Fe^3+^ ions, regardless of their location in octahedral or tetrahedral sites.

## Introduction

Copper ferrite is a ceramic material belonging to a class of metal-oxides of generic formula MFe_2_O_4_ where M represents a bi-valent transition metal ion, e.g. Mn^2+^, Ni^2+^, Co^2+^, Cu^2+^ ^1^, possessing the spinel structure.

Spinel ferrites in form of nanoparticles have received increasing attention mainly because of their magnetic and catalytic properties^2^ which are different from their parent bulk^3^ and are dependent on the size of the nanoparticles and the cation distribution within the structure^4–7^. In particular, copper ferrite nanoparticles have been shown to be suitable candidates for a wide range of technologically important applications, such as high energy storage materials^8,9^, magnetically recoverable catalysts^10^ for photocatalytic water splitting^11^ and as gas sensors^12^.

Many methods for the synthesis of copper ferrite nanoparticles have been proposed so far, the most common include high temperature solution phase approaches^13^, thermal decomposition of nitrates^14^, co-precipitation^15^, combustion process^16^ and sol-gel auto-combustion^17,18^.

However, these methods yield nanoparticles with average dimensions of several tens of nanometres and a poor degree of size and shape homogeneity. This is likely due to the aggregation and coalescence of the nanoparticles under the required thermal treatments. As an example, the sol-gel autocombustion process proposed by Ansari *et al*. for the synthesis of lead and copper hexaferrite (PbFe_12_O_19_ and CuFe_12_O_19_ respectively) using maltose as reducing agent^19,20^ enables to tune the size and shape of the produced nanoparticles by control of the calcination temperatures and the maltose content. Spherical nanoparticles with sizes of about 20 nm were obtained, but their TEM characterization clearly shows their tendency to agglomerate, which is a typical problem encountered for unsupported nanoparticles.

On the other hand, the control of the particle size has a major technological importance, because the magnetic and catalytic properties of nanoparticles are highly size dependent^16^.

The confined crystallisation of nanoparticles inside an amorphous matrix such as silica is a successful method for achieving the control of crystal growth while at the same time avoiding coalescence and growth. Previously, our group synthesised nanocomposites where cubic Mn, Ni, Co and Zn ferrite nanoparticles with average dimensions ranging from 5 to 10 nm are dispersed within the highly porous matrix of a silica aerogel^21,22^. In these nanocomposites, the nanoparticles are kept apart by the very highly porous silica matrix, avoiding their aggregation under thermal treatments, yielding nanoparticles with a high degree of size and shape homogeneity.

The ability to control the growth of ferrite nanoparticles has been used in this paper to investigate if the crystalline structure of copper ferrite is different at the nanoscale with respect to the bulk and if it is influenced by the presence of a silica matrix of variable density and texture. In particular, copper ferrite nanocrystals have been grown by confined crystallization in either a very low density silica aerogel matrix or a denser xerogel matrix.

Most of spinels have a cubic unit cell belonging to the *Fd-3m* space group, in which 24 metal ions are distributed in 8 tetrahedral and 16 octahedral sites within the close-packed arrangement made up of 32 oxygen ions. When the tetrahedral sites are entirely occupied by the bi-valent cation, the spinel is referred to as ‘normal’. When the bi-valent cations are located only in octahedral sites, the spinel is called ‘inverse’. Identifying with A and B the tetrahedral and octahedral sites respectively, the generic formula of a ferrite can be written as follows^23^:

$${[{{{\rm{M}}}^{2+}}_{1-{\rm{i}}}{{\rm{Fe}}}_{{\rm{i}}}^{3+}]}^{{\rm{A}}}{[{{\rm{M}}}_{{\rm{i}}}^{2+}{{\rm{Fe}}}_{2-{\rm{i}}}^{3+}]}^{{\rm{B}}}{{\rm{O}}}_{4-{\rm{\alpha }}}$$

with ‘i’ the inversion degree of the spinel and α the concentration of oxygen vacancies, if any. Many spinels undergo distortion upon ordering at low temperature, with the formation of a tetragonal *I4I/amd* unit cell.

Bulk copper ferrite is reported in the literature as an inverse spinel (i ≈ 1), which crystallises in either the tetragonal *I4I/amd* phase or the *Fd-3m* cubic one^24^. The tetragonal copper ferrite (t-CuFe_2_O_4_) is stable at room temperature and a transition to the cubic phase (c-CuFe_2_O_4_) is observed around 360–420 °C, where the two phases coexist, with a minor change in the inversion degree^16,25^. It was found that the tetragonal to cubic phase transition is accompanied by the reduction of part of Cu^2+^ to Cu^+^ ions, forming an oxygen deficient copper ferrite^26,27^. This evidence is supported by the possibility to selectively synthesise c-CuFe_2_O_4_ by performing a high temperature thermal treatment and the subsequent cooling process under inert atmosphere^28^.

In copper ferrite the tetragonal phase arises from the distortion along one of the axis of the octahedrons, caused by the presence of Cu^2+^ ions (d^9^), which impose the typical Jahn-Teller distortion, by the removal of the e_g_ orbital degeneracy^29,30^. Because the copper and iron cations share the octahedral sites in the spinel structure of the t-CuFe_2_O_4_, the unit cell is usually described with the Fe^3+^ ions having the same distortion, although there is no crystal-field stabilisation energy associated with the distortion at the Fe^3+^ (d^5^) sub-lattice.

In the literature, a considerable amount of work is found on the crystal structure of copper ferrite, where techniques such as X-ray diffraction (XRD), neutron diffraction and Mössbauer spectroscopy were mainly used^5,24,25^. However, diffraction techniques provide only an average structure and while Mössbauer spectroscopy can be used to study the inversion degree in the spinel structures^31^, it is not useful to study the copper environment that is essential to study the distortion produced in the tetragonal phase of copper ferrite by the presence of the Cu^2+^ ions.

X-ray absorption fine structure (XAFS) has the ability to study selectively and independently the Cu^2+^ and Fe^3+^ sites and has demonstrated to be exceptionally successful in studying ferrite structures^32–37^ as XAFS is element-specific and sensitive to the coordination environment of each specific absorbing atom. In the case of copper ferrite, the use of XAFS has been so far quite limited with the most comprehensive study being by Krishnan *et al*.^38^ where a structural investigation on CuFe_2_O_4_ nanoparticles was performed. Their Extended X-ray Absorption Fine Structure (EXAFS) and X-ray Absorption Near Edge Structure (XANES) results revealed that the cation distribution of copper ferrite nanoparticles is analogue to that of a bulk sample and all the Cu^2+^ ions occupy octahedral positions (complete inverse spinel). However, limited structural information was achieved on the local environment of Fe^3+^ ions since their EXAFS fit at the Fe K-edge did not distinguish between ions located in octahedral and tetrahedral sites.

In this paper XAFS has been used to study in detail the structure of CuFe_2_O_4_ in a bulk sample and in two nanocomposites where CuFe_2_O_4_ nanoparticles have been grown in either a very low density silica aerogel matrix or a denser xerogel matrix. The results clearly evidence for the first time that the accepted crystalline structure for t-CuFe_2_O_4_ based on diffraction methods lacks specific details, with the Jahn-Teller distortion in reality only affecting the copper ions.

## Methods

### Synthesis of the CuFe_2_O_4_ − SiO_2_ nanocomposites

#### Sol-gel procedure

The synthesis of the CuFe_2_O_4_ − SiO_2_ nanocomposites has been carried out following a 2-step catalysed sol-gel procedure previously published by our group^22^, which was successful in producing silica-based multicomponent gels containing a metal ferrite as dispersed phase, leading to high-quality MFe_2_O_4_ − SiO_2_ aerogels, where M = Mn, Ni, Co, Zn^21,31^. In this paper this synthetic protocol was used for the first time to produce both an aerogel and a xerogel nanocomposite with the same composition.

Briefly, in the first step an ethanolic solution of the metal salts (0.2299 g, 9.69∙10^−4^ mol, of Cu(NO_3_)_2_∙2.5H_2_O, Aldrich, 98%, 0.7985 g, 1.94∙10^−3^ mol, of Fe(NO_3_)_3_∙9H_2_O, Aldrich, 98%, 7.5 mL of absolute ethanol, Fisher Chemicals) was added to the pre-hydrolysed TEOS (7.9 mL, Aldrich, 98%) under acidic catalysis. The amounts of the precursors were calculated in such a way to produce a nanocomposite containing 10 wt.% CuFe_2_O_4_/(CuFe_2_O_4_ + SiO_2_). A solution containing urea (3.513 g of urea, NH_2_CONH_2_ Aldrich, >99%, 9 mL of absolute ethanol, Fisher Chemicals, 4.92 mL of distilled water) was then added under reflux for 128′ at 85 °C, in the second step to modulate pH achieving a fast gelation but at the same time avoiding the precipitation of metal hydroxides. The obtained sol was poured in cylindrical vials, which were sealed and kept at 40 °C until gelation, which took place within 20 h.

#### Aerogel nanocomposite

In order to obtain a CuFe_2_O_4_ − SiO_2_ aerogel, the solvent was removed from the alcogel in the supercritical state. To do so, the alcogel was inserted in a Parr 300 mL stainless-steel autoclave together with 70 mL of absolute ethanol. The autoclave was sealed and flushed with pure N_2_ to ensure an inert atmosphere. The autoclave was heated up to a temperature of 330 °C when the corresponding pressure of 70 atm was achieved, ensuring that the solvent was in the supercritical state. The autoclave was then slowly vented at constant temperature and the aerogel was obtained.

#### Xerogel nanocomposite

A CuFe_2_O_4_ − SiO_2_ xerogel was obtained by submitting the alcogel to slow evaporation of the solvent in an open container, at 40 °C for 70 h until constant weight.

#### Thermal treatments

After drying, the aerogel and xerogel samples were powdered and submitted to the same calcination treatments, performed in static air. The dried gels were calcined first at 450 °C for 1 h, to remove organics, and then at 750 °C for 1 h, 750 °C for 6 h and 900 °C for 1 h to promote the growth of the copper ferrite nanoparticles. The formation of the CuFe_2_O_4_ is complete only under thermal treatment at 900 °C, as previously observed in other ferrite-silica nanocomposites^21,22,31,33,35,36^. The samples will be named hereafter as ACuFe and XCuFe to indicate the aerogel and xerogel respectively.

#### Synthesis of bulk – CuFe_2_O_4_

The synthesis of a bulk sample of CuFe_2_O_4_ was performed following a procedure already reported in the literature^39^. According to this procedure, appropriate amounts of metal nitrates were dissolved in water and the solution was heated at 95 °C up to dryness. The resulting solid was calcined first at 150 °C, allowing for the complete decomposition of nitrates. Further calcination at 900 °C for 8 h followed by slow cooling in static air is needed to promote the formation of tetragonal copper ferrite. The sample will be named hereafter as bulk–CuFe_2_O_4_.

#### Characterization of the nanocomposites

X-ray Diffraction (XRD) patterns were recorded on a Panalytical Empyrean diffractometer equipped with a graphite monochromator on the diffracted beam and a X’Celerator linear detector. The scans were collected within the range of 10–90° (2θ) using Cu Kα radiation. The average size of crystallite domains was calculated using the Scherrer equation, t = 0.91 λ/(B cos θ), where t is the crystallite size, λ is the incident radiation wave-length, θ is the Bragg angle and B is the full-width at half-maximum of the diffraction peak, corrected for instrumental broadening using a standard LaB_6_ sample^40^.

Transmission electron microscopy (TEM) images were recorded on a Hitachi H-7000 instrument running at 125 kV and equipped with an AMT DVC (2048 × 2048 pixel) CCD Camera. Prior to observation, the samples were finely ground and deposited on a carbon-coated copper grid. SEM images were recorded on a Hitachi S4700 FEG-SEM.

The X-ray absorption spectra were recorded on the B18 beamline at the DIAMOND synchrotron (Oxfordshire, UK). XANES and EXAFS spectra at the Fe (7112 eV) and Cu (9790 eV) K-edge were collected at room temperature in transmission mode using a Si(111) monochromator. The monochromator energy scale was calibrated via a third ion chamber with Fe/Cu foils. The stability of the energy scale was ±0.1 eV. The samples, in form of powder, were diluted with polyvinylpyrrolidone (PVP) in an appropriate concentration and pressed to form a pellet. The data analysis was performed using the ATHENA and ARTEMIS software^41^. With ATHENA, the absorption edge, E_0_, is determined, and the absorption due to the isolated atom is subtracted, by fitting the pre-edge and post-edge regions to obtain χ(k). ATHENA is also used to process the XANES data. The software ARTEMIS is used to perform the fit of the EXAFS region to scattering models in R-space obtained by FEFF, validated on standard compounds. The number of fitted parameters was always lower than the number of independent points.

## Results and Discussion

**Figure 1:**
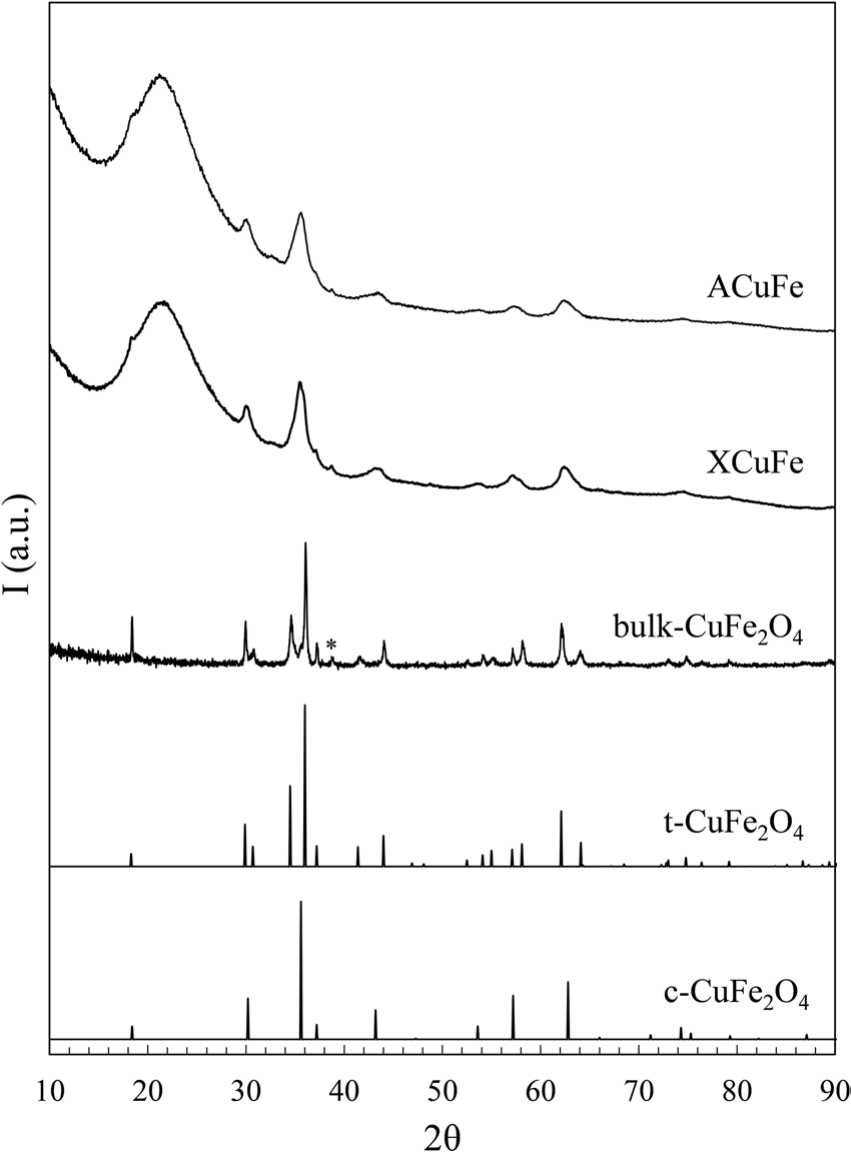
XRD patterns of ACuFe, XCuFe and bulk-CuFe_2_O_4_. The pattern of the tetragonal and cubic phases of copper ferrite are also reported. *Represents a peak identified as a small impurity of CuO^48^.

In Fig. 1, the XRD patterns of the ACuFe and XCuFe are shown, together with the bulk sample. The reference patterns of the tetragonal and cubic CuFe_2_O_4_ crystalline phases are also shown as a refs^42,43^. The pattern of the bulk sample can be unambiguously identified as corresponding to the tetragonal phase. In fact, the peaks at 2θ values of ~31°, 34.5° and 41.4°, found in the XRD pattern of the bulk sample, can only be assigned to the tetragonal phase. Likewise, some peaks corresponding only to the cubic phase, such as one at 2θ = 43.2° were not found in the XRD pattern of the bulk-CuFe_2_O_4_.

The patterns of the aerogel and xerogel samples show the typical halo due to the amorphous silica matrix and the superimposed broad peaks corresponding to nanocrystalline copper ferrite. Due to the broadening of the peaks, it is not possible to determine if the copper ferrite nanoparticles are in the tetragonal or cubic crystalline phase. The average sizes of the crystallites, determined applying the Scherrer equation to the most intense peak at ∼35° and correcting for instrumental broadening, are 6 nm in the case of the aerogel and 7 nm in the case of the xerogel sample. The typical error on the determination of the average crystal size is ± 1 nm.

The XRD characterization of the nanocomposites, as a function of the calcination temperature, is reported in Figures S1 and S2, Supp. Info. The XRD patterns show that the peaks corresponding to the CuFe_2_O_4_ crystalline phase appear only upon calcination at 750 °C for 1 h. However, the formation of the CuFe_2_O_4_ phase is only complete after a thermal treatment of 900 °C as inferred by inspecting the relative intensity of the peaks. These results are also in agreement with previous findings in other ferrite-silica nanocomposites^21,22,31,33,35,36^.

**Figure 2:**
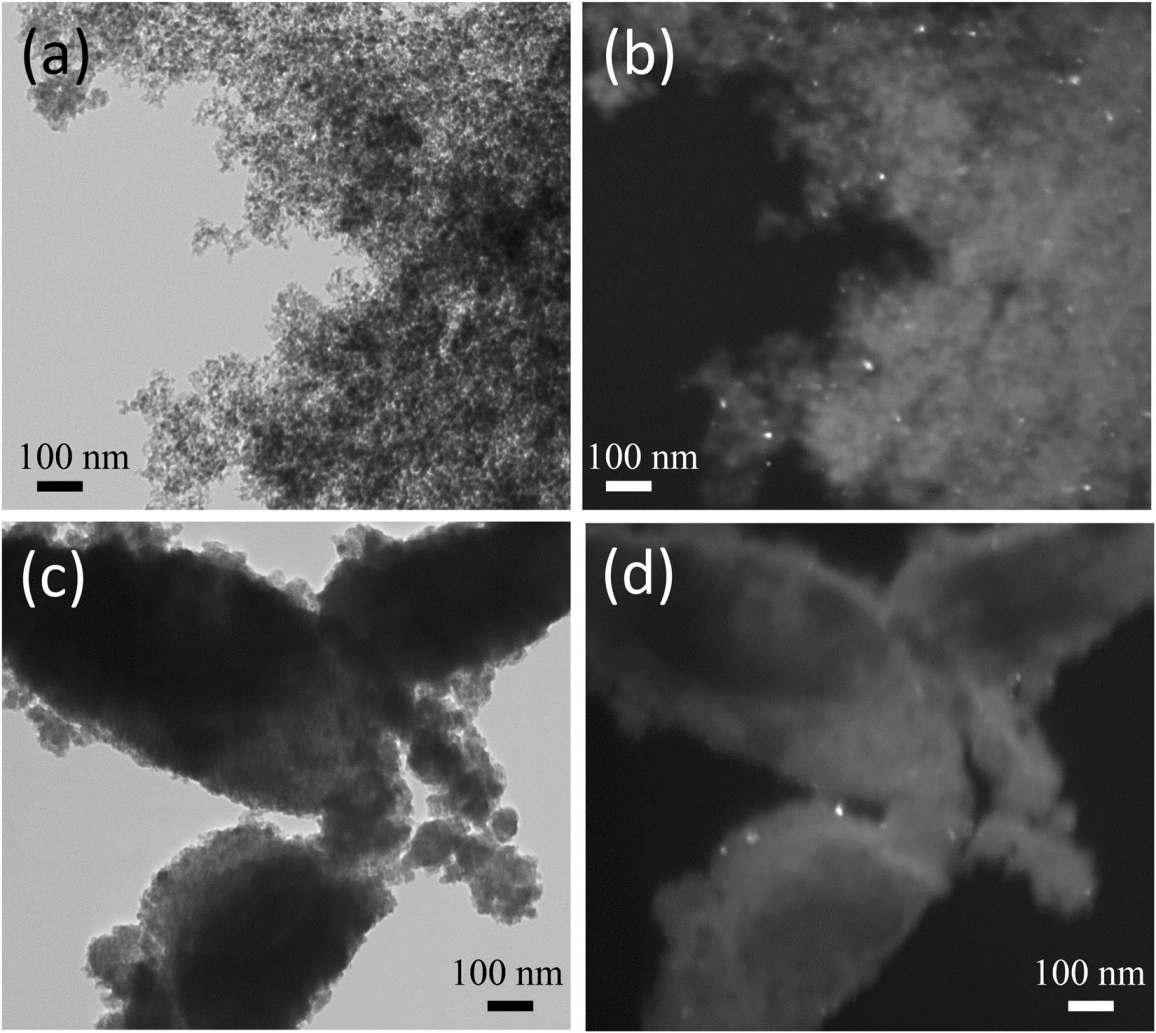
TEM images of the ACuFe (**a**,**b**) and XCuFe (**c**,**d**) nanocomposites; bright field on the left, dark field on the right.

In Fig. 2, bright field and dark field images of the aerogel and xerogel samples are shown. The TEM images in bright field mode shows the highly porous texture typical of the aerogel, Fig. 2(a), while the xerogel sample appears denser and less porous, as expected, Fig. 2(c). This difference in porosity has also been confirmed by N_2_-physisorption reported in the SI, with the xerogel having a much lower pore volume and reduced surface area with respect to the aerogel. By looking at the dark field TEM images in Fig. 2(b) and (d), the copper ferrite dispersed nanophase can be seen as bright spots on top of the darker background due to the silica matrix. The nanoparticles appear uniformly dispersed within the porous matrix. In the case of the xerogel (Fig. 2(d)), due to the denser silica matrix, the nanocrystallites are only visible on the edges of the particles. The average size of the nanocrystals is in agreement with the values determined from the XRD patterns, confirming that the Scherrer equation was able to provide the correct value. The size of the crystals is similar in the two composites despite their very different texture and porosity, indicating the growth of nanocrystals within the matrix is driven by the thermal treatment of the gels.

**Figure 3:**
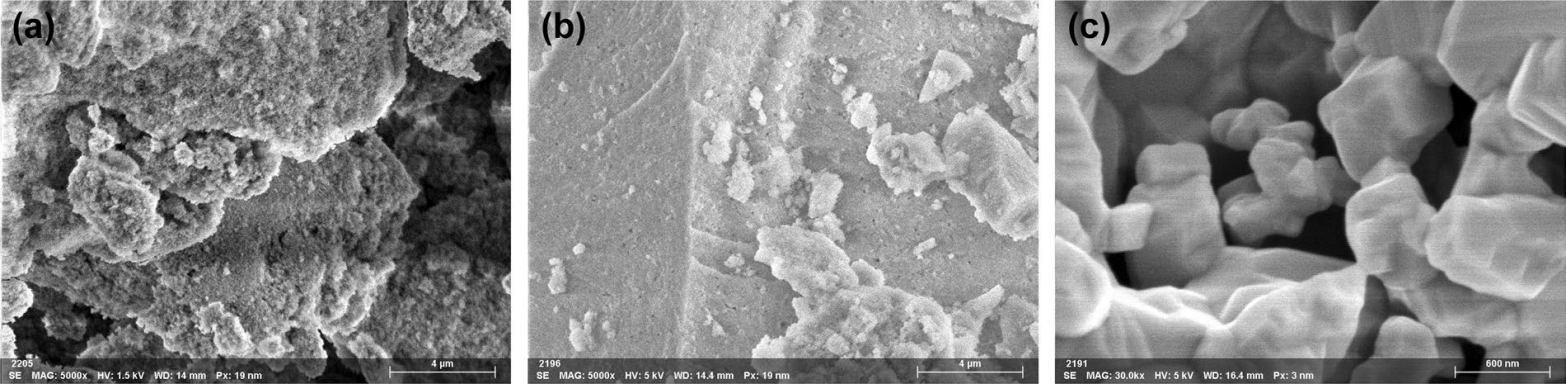
FESEM images of the ACuFe (**a**), XCuFe (**b**) and bulk-CuFe_2_O_4_.

FESEM investigation was used to gain insights on the texture and compositional homogeneity of the materials. The images reported in Fig. 3 for the aerogel, xerogel and bulk samples support the results obtained by TEM and physisorption investigation. In particular, the highly porous texture of the aerogel, the denser features in the xerogel due to the occurrence of smaller pores and the microcrystalline aspect of the bulk ferrite sample are observed.

### Symmetry and coordination at the metal sites

**Figure 4:**
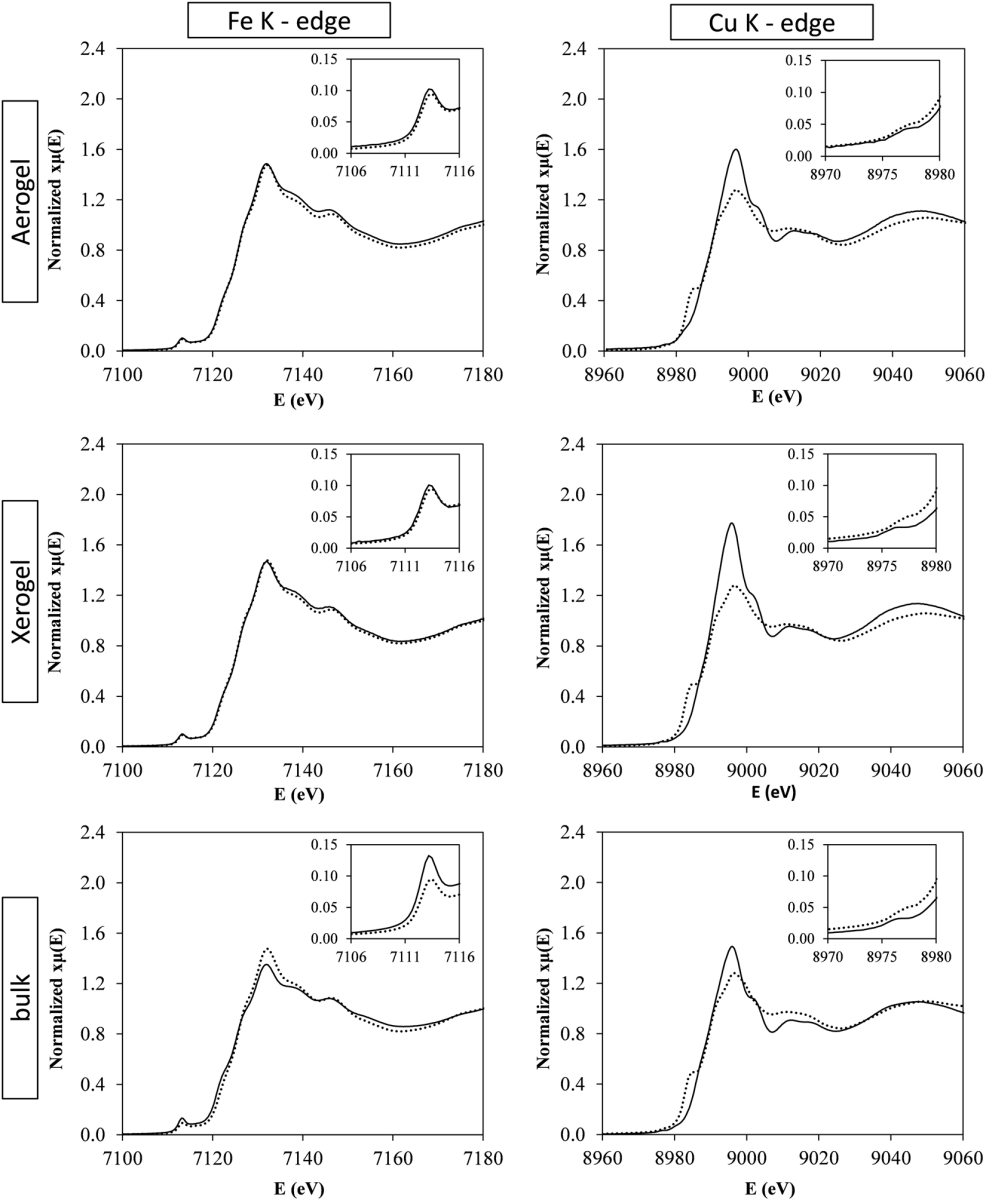
XANES spectra of ACuFe, XCuFe, and bulk-CuFe_2_O_4_, at the Fe and Cu K-edge. Bold line: samples; dots: γ-Fe_2_O_3_ in the case of Fe K-edge, CuO in the case of Cu K-edge. The pre-peak region is enlarged in the inset.

XAFS spectroscopy has been used for the characterization of the ACuFe, XCuFe and the bulk sample. The analysis of the XANES region gives information on the oxidation state of the absorbing atom and on its coordination geometry. In Fig. 4, the XANES spectra of the samples are reported, at both the Cu and Fe K-edges. At the Fe K-edge, the spectrum of γ-Fe_2_O_3_ (maghemite) reference compound is reported superimposed to those of the samples while, at the Cu K-edge, the absorption spectrum of CuO (tenorite) reference compound is reported.

In Table 1 the absorption edges (E_0_) and the corresponding oxidation states are reported. By comparing the position of the absorption edges with reference compounds the oxidation states are Fe^3+^ and Cu^2+^ for all samples^37^.

**Table 1:** Absorption energies and oxidation states for the samples and reference compounds at both the Cu and Fe K-edge.

Sample	Element	Oxidation state	E_0_ (eV)
α−Fe_2_O_3_	Fe	+3	7125.5
γ−Fe_2_O_3_	Fe	+3	7125.5
CuO	Cu	+2	8989.1
ACuFe	Cu	+2	8989.8
	Fe	+3	7125.5
XCuFe	Cu	+2	8990.1
	Fe	+3	7125.5
Bulk	Cu	+2	8989.9
	Fe	+3	7125.5

A close inspection of the pre-edge region, enlarged in the insets of Fig. 4, provides qualitative information on the coordination geometry of the absorbing atom. In this region of the spectra, a pre-peak might be present, due to electronic transition involving the 3d orbitals^44^. The intensity of the peak is sensitive to the local environment of the absorbing atom, and is more intense in the presence of non-centrosymmetric coordination geometry, e.g. when the cation is located in a tetrahedral site, as in γ-Fe_3_O_3_. A well-defined pre-peak is present in the case of the Fe K-edge for both the nanocomposites and for the bulk sample, which is indicative of the presence of Fe^3+^ cations in tetrahedral sites. In the case of the Cu K-edge, the pre-peak has a very low intensity, in both CuO and CuFe_2_O_4_. Note that Cu^2+^ has a d^9^ configuration and the pre-edge peak is due to 1s-3d transitions with an overwhelming 1sd^9^ configuration^45^. As in other transition metal K-edges, the Cu pre-edge peak intensity is sensitive to the degree of centrosymmetry of the Cu site^46^, and is significantly larger for tetrahedral coordination (e.g. in CuAl_2_O_4_ and CuCr_2_O_4_) and smaller for octahedral coordation (e.g. CuO and CuFe_2_O_4_)^47^. The similar, low pre-edge peak intensities observed for CuO and for the CuFe_2_O_4_ samples in Fig. 4 suggest that all the Cu^2+^ cations are located in octahedral sites. The analysis of the pre-peak region therefore suggests that the spinel is completely (or highly) inverted, as expected for CuFe_2_O_4_. We also note that the pre-edge and post-edge features of the xerogel and aerogel samples are very similar to those of the bulk, which suggests a close similarity of the iron and copper ions environment in the two nanocomposites and the bulk.

### Distortions at the Fe and Cu sub-lattices

**Figure 5:**
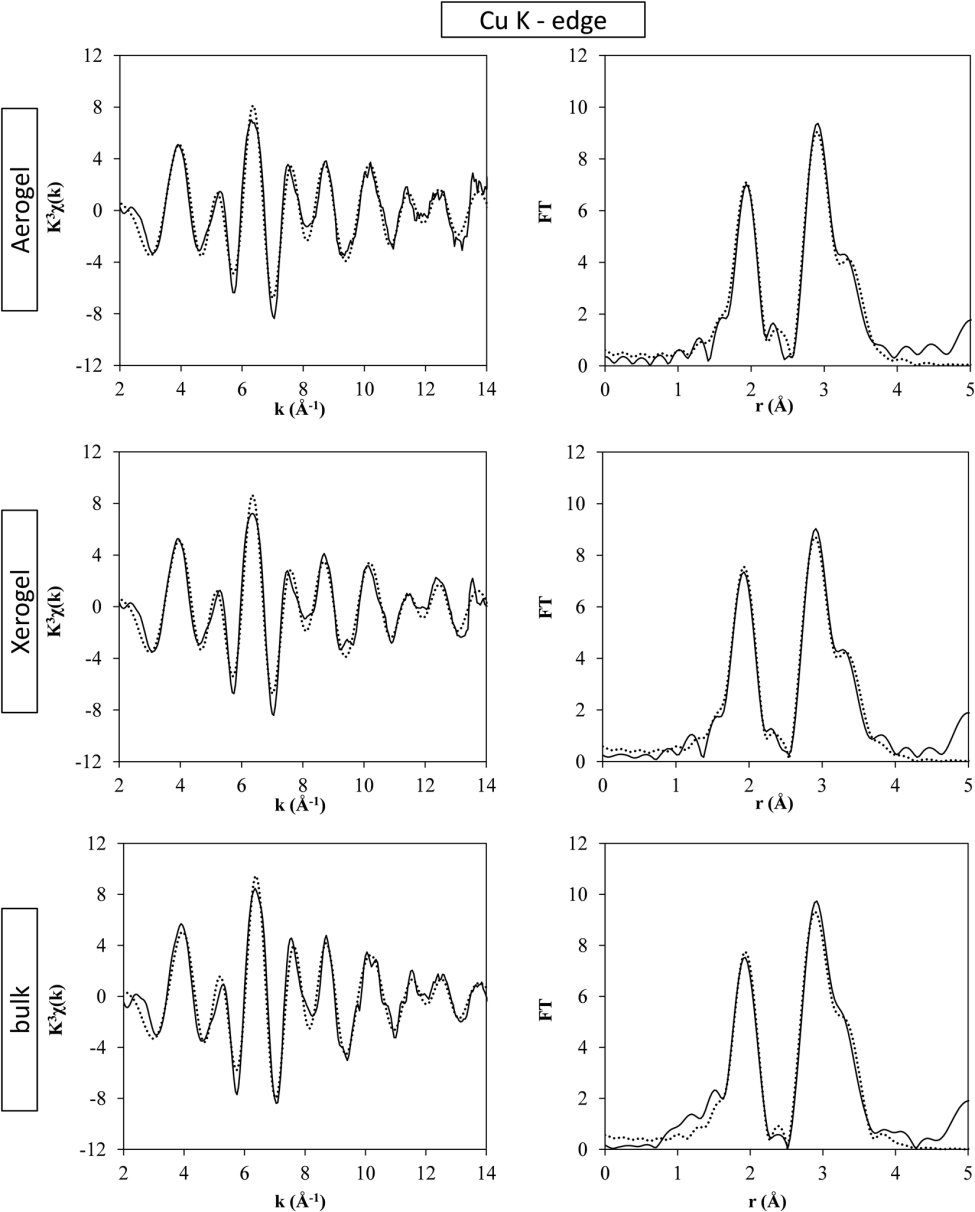
Fit of the EXAFS region at the Cu K-edge of the ACuFe, XCuFe and bulk-CuFe_2_O_4_ samples; k^3^χ(k) (left) and corresponding FTs (right). Full line: experiment; dots: fit.

**Figure 6:**
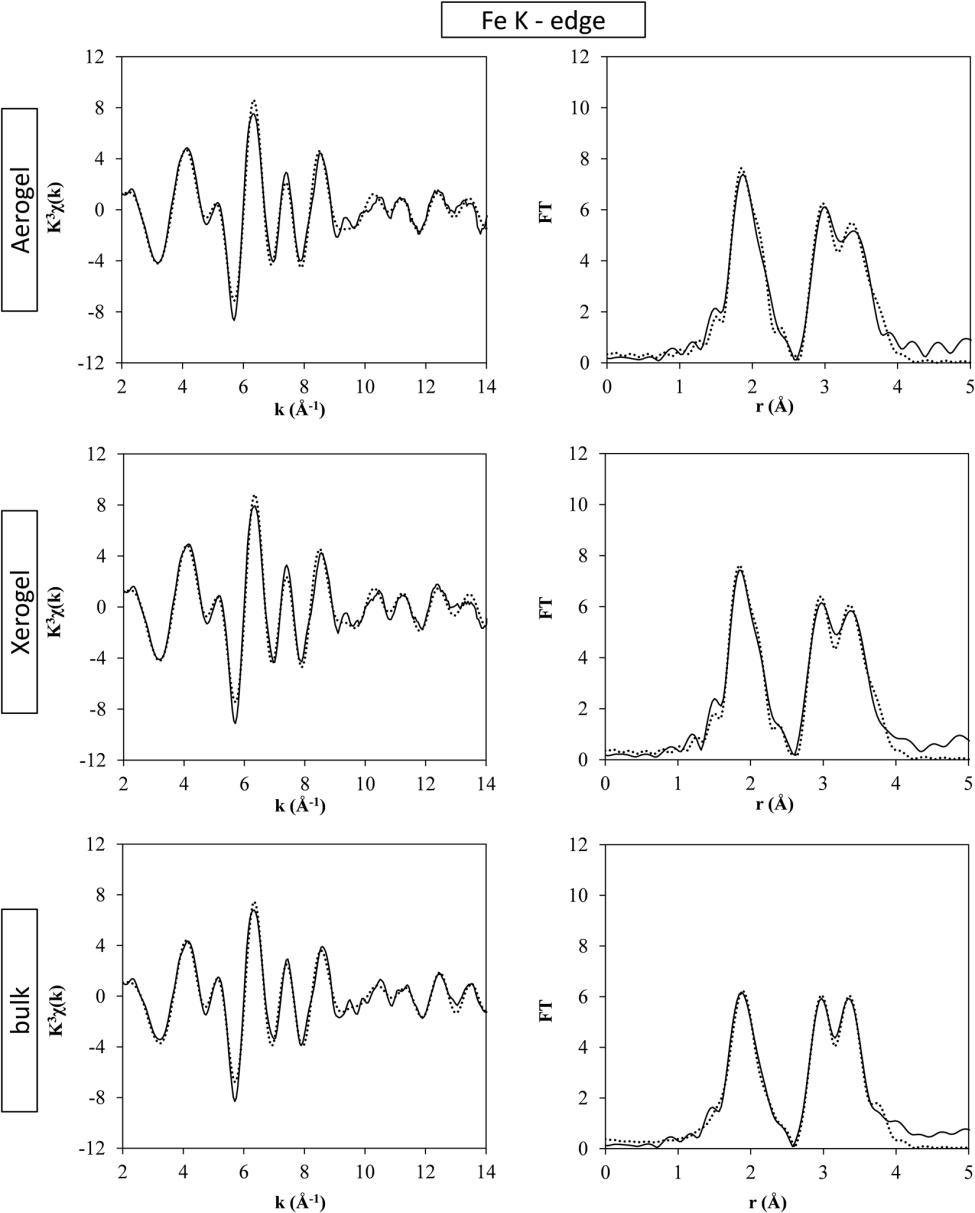
Fit of the EXAFS region at the Fe K-edge of the ACuFe, XCuFe and bulk-CuFe_2_O_4_ samples; k^3^χ(k) (left) and corresponding FTs (right). Full line: experiment; dots: fit.

The analysis of the EXAFS region at both the Cu and Fe K-edges allows obtaining in depth and quantitative insights on the coordination environment of each metal ion within the crystal structure, including the distances of each coordination shell. In Fig. 5, the k^3^χ(k) and the corresponding Fourier Transforms (FTs) are reported for the nanocomposites and the bulk sample at the Cu K-edge. The same is reported for the Fe K-edge in Fig. 6.

First of all, the qualitative analysis of the FTs can give further confirmation regarding the inversion degree of the spinel. At both the Cu and Fe K-edges, the first peak is generated by the distances between the absorbing atom and the first coordination shell of oxygen atoms. The second peak is mainly due to distances between the absorber with either copper or iron. In particular, two main unresolved contributions can be identified: one centred around 2.9 Å and the second centred around 3.5 Å. In the spinel structure, 2.9 Å corresponds to the distance between two octahedral sites (M_B_-M_B_) while the distances involving tetrahedral sites (M_B_-M_A_, M_A_-M_B_ and M_A_-M_A_) are all centred at about 3.5 Å. By looking at the FTs at the Cu K-edge, Fig. 5, the peak centred at 2.9 has the highest intensity suggesting that the majority of the Cu^2+^ ions are located in octahedral sites. The much less intense peak around 3.5 Å is therefore likely due to distances between the Cu^2+^ absorbing ions with Fe^3+^ backscattering ions, located in tetrahedral sites. The same features were found in a previous work, in the case of Ni ferrite, which is an inverse spinel^32^. In the case of the Fe K-edge, the iron ions seem to be equally distributed between octahedral and tetrahedral sites, since the contribution at 2.9 Å due to M_B_-M_B_ distances and the one at 3.5 Å due to M_A_-M_B_, M_B_-M_A_ and M_A_-M_A_ distances have comparable heights.

Quantitative information was obtained performing the fitting of the EXAFS region, after carefully choosing the theoretical model. In all the best fitting only single scattering paths were added after checking that the contribution of multiple scattering paths was less significant. A first attempt in fitting the data at the Cu K-edge employing a model based on the cubic copper ferrite crystalline phase did not give satisfactory results on neither of the samples. This was not surprising in the case of the bulk sample, as XRD clearly shows that the bulk sample is in the tetragonal phase and the cubic model does not take into account the Jahn-Teller distortion, whose effect is strongest on the first coordination shell.

A good fit of the EXAFS region at the Cu K-edge was instead obtained for the bulk sample and for the nanocomposites using the same theoretical model based on the tetragonal copper ferrite crystalline phase, with inversion degree i = 1, which takes into account the effect of the Jahn-Teller distortion. Nine coordination shells, up to a distance of 4 Å, were included in the fitting. Coordination numbers were kept fixed, given by the crystal structure, while the distances (ΔR_i_), the Debye-Waller factors (σ^2^) and ΔE_0_ were left free to vary. S_0_^2^ was determined by fitting the data of the bulk sample, and then kept fixed for the nanocrystalline samples. The crystal structure of tetragonal copper ferrite requires that the M_B_-M_B_ distances are split in two contributions, since the octahedral sites are occupied by both Cu^2+^ and Fe^3+^ ions with 50% occupancy. However, this does not increase the number of fitted parameters because the same distances (ΔR_i_) and Debye-Waller factors (σ^2^) are imposed throughout the fitting. The Cu-O distances at R > 3.5 Å and the corresponding Debye Waller factors were kept fixed, due to their small weight. The results of the fit are very similar for all the samples, indicating that the copper environment is typical of the tetragonal phase also for the xerogel and aerogel samples, where the XRD results where ambiguous, due to peak broadening. Best fit parameters are shown in Table 2 for the aerogel nanocomposite and those for the bulk and the xerogel samples are reported in Tables S2 and S3 in the SI.

**Table 2:** Best fit parameters obtained by fitting the experimental EXAFS of the ACuFe sample at the Cu K-edge with a 9 shell model of tetragonal copper ferrite. Coordination numbers (N), interatomic distances (R), Debye-Waller factors, S_0_^2^, ΔE_0_ and R-factor are shown.

ACuFe		Cu K-edge		
Abs.-Backscatter	N	σ^2^(Å^2^)	R(Å)	Occupancy
Cu-O	4.0	0.007 ± 0.001	1.97 ± 0.01	1.0
Cu-O	2.0	0.009 ± 0.003	2.22 ± 0.02	1.0
Cu-Cu_B_^*^	2.0	0.005 ± 0.001	2.90 ± 0.01	0.5
Cu-Fe_B_^*^	2.0	0.005 ± 0.001	2.90 ± 0.01	0.5
Cu-Cu_B_^**^	4.0	0.010 ± 0.003	3.00 ± 0.02	0.5
Cu-Fe_B_^**^	4.0	0.010 ± 0.003	3.00 ± 0.02	0.5
Cu-Fe_A_	4.0	0.010 ± 0.002	3.44 ± 0.03	1.0
Cu-Fe_A_	2.0	0.012 ± 0.006	3.65 ± 0.06	1.0
Cu-O	2.0	0.040	3.557	1.0
Cu-O	4.0	0.040	3.633	1.0
Cu-O	2.0	0.040	3.705	1.0
**S**_**0**_^**2**^ = 0.9			**ΔE**_**0**_ = 0.00 ± 1.4 eV	
**R-factor** = 0.021				

^*^The distances involving the Cu^2+^ absorber with the ions located in the tetrahedral sites are split into two contributions with the same interatomic distances and Debye-Waller factors, as described in the text. ^**^The distances involving the Cu^2+^ absorber with the ions located in the octahedral sites are split into two contributions with the same interatomic distances and Debye-Waller factors, as described in the text.

When performing the fit of the EXAFS region at the Fe K-edge the same theoretical model corresponding to the tetragonal phase which was used for fitting the Cu K-edge, did not produce satisfactory results. The best fit was obtained instead using the model of the cubic copper ferrite crystalline phase, keeping the inversion degree i = 1. Because of the inversion the Fe^3+^ absorbing ions must be split into two series of contributions, scaled to 50% occupancy, the first involving Fe^3+^ in the octahedral sites and the second involving the Fe^3+^ in tetrahedral sites respectively. Distances up to 4 Å were fitted, which includes 4 shells to fit the contributions due to Fe^3+^ in the tetrahedral sites and 5 shells to fit the contributions due to Fe^3+^ in the octahedral sites. Because of the crystal structure of cubic copper ferrite, several paths need to be split in the fitting. However, this does not overcomplicate the fitting because the same distances (ΔR_i_) and Debye-Waller factors (σ^2^) are imposed throughout the fitting for any distance which is the same from a crystallographic point of view. In particular, the distances with backscattering ions in octahedral sites (M_A_-M_B_ and M_A_-M_B_) need to be split because they are involving both Fe^3+^ and Cu^2+^, scaling the occupancy of each contribution by a further 50% while imposing the same distances (ΔR_i_) and Debye-Waller factors (σ^2^). Moreover, the Fe_A_-Fe_B_ and Fe_B_-Fe_A_ contributions represent the same distance and therefore the additional constraint of using the same ΔR_i_ and σ^2^ was used. Finally, the same ΔR_i_ and σ^2^ were used to fit two distances at 3.6 Å (Fe_A_-O and Fe_B_-O), since they are too close to be distinguished by EXAFS, even if they are different from a crystallographic point of view.

Also in the case of the Fe K-edge the results of the fit are very similar for all samples. The best fit parameters are shown in Table 3 for the aerogel nanocomposite and those for the bulk and xerogel samples are reported in Tables S4 and S5 in the SI.

**Table 3:** Best fit parameters obtained by fitting the experimental EXAFS of the ACuFe sample at the Fe K-edge with a 4 shell model in the case of the tetrahedral sites and a 5 shell model in the case of the octahedral sites using a model of cubic copper ferrite. Coordination numbers (N), interatomic distances (R), Debye-Waller factors, S_0_^2^, ΔE_0_ and R-factor are shown.

ACuFe		Fe K-edge		
Abs-Backscatter	N	σ^2^(Å^2^)	R(Å)	Occupancy
Fe_A_-O	4.0	0.001 ± 0.001	1.88 ± 0.01	0.50
Fe_A_-Fe_B_^*^	12.0	0.009 ± 0.003	3.45 ± 0.04	0.25
Fe_A_-Cu_B_^*^	12.0	0.009 ± 0.003	3.45 ± 0.04	0.25
Fe_A_-O^***^	12.0	0.01 ± 0.06	3.6 ± 0.1	0.50
Fe_A_- Fe_A_	4.0	0.006 ± 0.005	3.6 ± 0.1	0.50
Fe_B_-O	6.0	0.003 ± 0.001	2.00 ± 0.01	0.50
Fe_B_-Fe_B_^**^	6.0	0.007 ± 0.001	2.98 ± 0.01	0.25
Fe_B_-Cu_B_^**^	6.0	0.007 ± 0.001	2.98 ± 0.01	0.25
Fe_B_-Fe_A_^*^	6.0	0.009 ± 0.003	3.45 ± 0.04	0.50
Fe_B_-O^***^	2.0	0.01 ± 0.6	3.6 ± 0.1	0.50
Fe_B_-O	6.0	0.03 ± 0.3	3.65	0.50
**S**_**0**_^**2**^ = 0.7			**ΔE**_**0**_ = −2.64 ± 0.73 eV	
**R-factor** = 0.013				

^*^The distances involving the Fe^3+^ absorber in the tetrahedral site with the ions located in the tetrahedral sites, and the distances involving the Fe^3+^ absorber in the octahedral site with the Cu^2+^ located in the tetrahedral sites, are split in contributions with the same interatomic distances and Debye-Waller factors, as described in the text. ^**^The distances involving the Fe^3+^ absorber in the octahedral site with the ions located in the octahedral sites are split into two contributions, as described in the text. ^***^The distances and Debye-Waller factors involving two Fe**-**O distances which are intrinsically different from a crystallographic point of view, but too close to be distinguished by EXAFS, were kept to the same values, as described in the text.

The cooperative Jahn-Teller distortion, occurring at the octahedral sites occupied by the Cu^2+^ ions, causes the formation of the tetragonal phase. The fit of the EXAFS region at the Cu K-edge confirms that the six Cu-O distances are splitted into 4 short Cu-O distances and two longer ones. However, the octahedral sites within the unit cell are 50% occupied by Cu^2+^ ions, with Fe^3+^ ions occupying the other 50% of the sites. By only using XRD it is not possible to discriminate whether the octahedral sites occupied by the Fe^3+^ ions are also affected by this change in symmetry. This is because XRD provides the average structural environment, and as a consequence the published crystal structures for tetragonal copper ferrite is not distinguishing between the two cations^24^. Instead, EXAFS spectroscopy allowed us to find clear evidence that all the six Fe-O distances in the first coordination shell of copper ferrite are identical (see Table 3) since a good fit at the Fe K-edge was only obtained by using a model based on the cubic crystalline phase. It should be noted that also when fitting only the first peak in the FTs the same results were obtained, i.e. best fit was obtained using the tetragonal copper ferrite phase at the Cu K-edge and the cubic copper ferrite phase at the Fe K-edge.

These results therefore unambiguously indicate that the Fe^3+^ ions define a cubic sub-lattice within the tetragonal lattice defined by the Cu^2+^ ions. This is likely possible because of the presence of a number of available interstitial sites within the spinel crystal structure, where the Fe^3+^ ions can migrate in order to avoid any axial distortion on their first coordination shell.

## Conclusions

The synthesis of t-CuFe_2_O_4_ nanocrystals with controlled features has been achieved by performing the crystal growth within the amorphous porous matrix of a silica aerogel and xerogel. The structural and textural features as assessed by TEM, XRD and N_2_-physisorption point out to the formation of t-CuFe_2_O_4_ nanophase with crystals sizes in the region of 6–7 nm and high size homogeneity, both in a relatively dense xerogel and in a highly porous aerogel.

EXAFS and XANES investigation carried out at the Fe and Cu K-edges indicates that the structure of the nanocrystalline samples is very similar to that of the bulk t-CuFe_2_O_4_. Very importantly, EXAFS spectroscopy has shed a new perspective on the crystalline structure of tetragonal copper ferrite, which is retained at the nanophase. In particular, two different sub-lattices are present within the structure, one tetragonal and one cubic, defined by Cu^2+^ and Fe^3+^ ions respectively. This is ascribed to the difference in the coordination environment of the two different cations located in the octahedral sites. In particular, the electronic structure of Cu^2+^ (d^9^) imposes the axial distortion of the octahedron, i.e. the Jahn-Teller distortion, while the coordination environment of the Fe^3+^ ions located in the remaining half of the octahedral sites remains unaffected and can therefore be regarded as a cubic sub-lattice. This demonstrates that the ability of studying the Cu and Fe local environment separately and independently is able to emphasize local structural features that diffraction techniques cannot achieve since they provide an average structure.

We believe that a detailed description of the oxidation state and coordination symmetry at the metal sites could provide a deeper understanding of the properties of copper ferrite and copper-based nanoparticles which have prospective technologically important applications as cost-effective non noble metal catalysts, high energy storage materials, and sensors.

## Electronic supplementary material

Supplementary Information
