# Supplementary material for: Evidence of a cubic iron sub-lattice in t-CuFe2O4 demonstrated by X-ray Absorption Fine Structure
Source: Sci Rep. 2018 Jan 15;8:797. doi: 10.1038/s41598-017-19045-8 (PMC5768695; doi:10.1038/s41598-017-19045-8)
Supplement: Supplementary file 1 — Supplementary Information [file 41598_2017_19045_MOESM1_ESM.pdf]

Evidence of a cubic iron sub-lattice in t-$CuFe_2O_4$ demonstrated by X-ray Absorption Fine Structure

Francesco Caddeo[a, †], Danilo Loche[a], Maria F. Casula[b] and Anna Corrias[a]*

[a] School of Physical Sciences, Ingram Building, University of Kent, Canterbury CT2 7NH, United Kingdom

[b] INSTM and Dipartimento di Scienze Chimiche e Geologiche, Università di Cagliari, S.S. 554, bivio per Sestu, Monserrato, CA, Italy

[†] Present Address: ZIK SiLi-nano, Martin-Luther-University Halle-Wittenberg, Karl-Freiherr-von-Fritsch-Straße 3, 06120 Halle (Saale), Germany

Supporting Information

XRD characterization

The formation of the copper ferrite nanophase within the silica matrix was promoted by heat treatment. Different thermal treatments were performed by varying the final temperature and the time the samples were kept at the final temperature. The XRD patterns of the aerogel and xerogel materials at different calcination conditions shown in Figure S1 and S2 respectively, indicate that the formation of the $CuFe_2O_4$ phase is only complete after a thermal treatment of 900 °C.

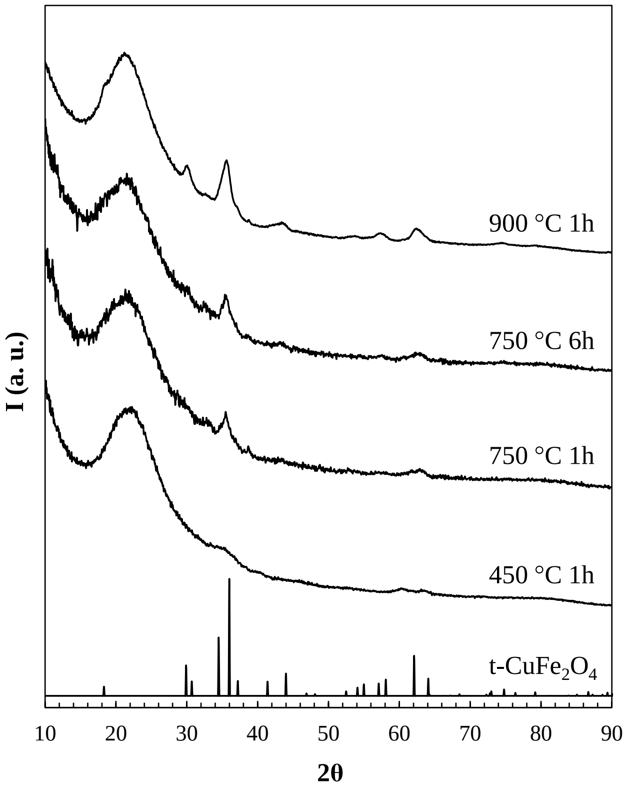

Figure S1. XRD patterns of ACuFe after thermal treatments at 450 °C for 1h, 750 °C for 1h, 750 °C for 6h and 900 °C for 1h. The pattern of the tetragonal phase of copper ferrite is also reported at the bottom of the figure.

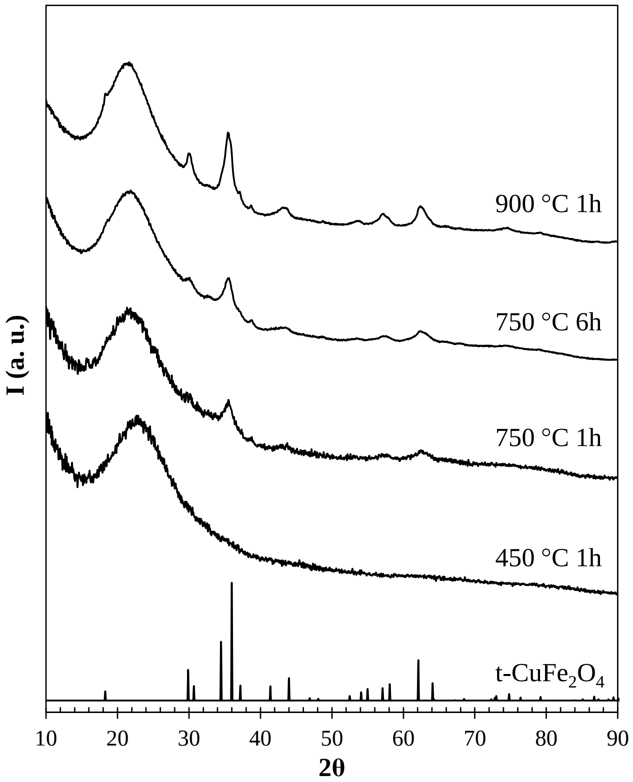

Figure S2. XRD patterns of XCuFe after thermal treatments at 450 °C for 1h, 750 °C for 1h, 750 °C for 6h and 900 °C for 1h. The pattern of the tetragonal phase of copper ferrite is also reported at the bottom of the figure.

N_2-physisorption measurements

Surface areas, pore sizes and pore volumes were obtained from N_2 adsorption-desorption measurements at 77 K recorded on a Micromeritics ASAP2020. Surface area was estimated using the Brunauer–Emmett–Teller (BET) model, pore size and pore volumes were estimated using the Barret–Joyner–Halenda (BJH) method.[S1,S2]

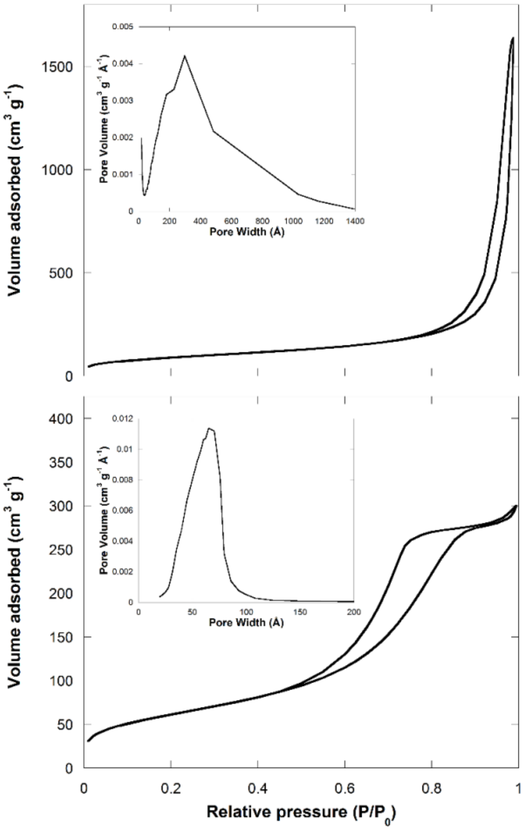

Figure S3 N_2 physisorption isotherms of the ACuFe (top) and XCuFe (bottom) samples and pore size distribution calculated using the desorption branch (inset).

The different textural features presented by the aerogel and xerogel samples is confirmed by N_2 physisorption measurements at 77 K (Figure S1, Table S1). The ACuFe sample exhibits a type IIb isotherm with an H3 hysteresis loop located at high values of relative pressures, due to interconnected mesopores with large size,[S3] typical of nanocomposite aerogels.[19-20] The XCuFe sample instead shows a type I reversible isotherm typical of a microporous texture.[S3] As shown in Table S1 the average pore diameters, determined by the BJH method, are 30 nm in the case of the aerogel while they are significantly smaller (7 nm) in the case of the xerogel. The xerogel has a lower surface area and a significantly lower pore volume, as expected.

Table S1 N_2-physisorption results for of the ACuFe and XCuFe nanocomposites. Surface area, S, pore volume, V_P, and pore diameter are reported.

Sample	S ($m^2 \cdot g^{-1}$)	V_P ($cm^3 \cdot g^{-1}$)	Pore diameter (nm)
ACuFe	325	2.53	30
XCuFe	224	0.47	6 - 7

EXAFS fit

Table S2 Best fit parameters obtained by fitting the experimental EXAFS of the bulk-$CuFe_2O_4$ sample at the Cu K-edge with a 9 shell model of tetragonal copper ferrite. Coordination numbers (N), interatomic distances (R), Debye-Waller factors, S_0^2, ΔE_0 and R-factor are shown.

**Bulk $CuFe_2O_4$**			**Cu K-edge**	
**Abs. – Backscatter**	**N**	**σ^2(Å^2)**	**R(Å)**	**Occupancy**
Cu-O	4.0	0.006 ± 0.001	1.97 ± 0.01	1.0
Cu-O	2.0	0.011 ± 0.004	2.24 ± 0.03	1.0
$Cu-Cu_B$	2.0	0.004 ± 0.002	2.86 ± 0.02	0.5
$Cu-Cu_B$	4.0	0.007 ± 0.003	2.98 ± 0.01	0.5
$Cu-Fe_B$	2.0	0.004 ± 0.002	2.86 ± 0.02	0.5
$Cu-Fe_B$	4.0	0.007 ± 0.003	2.98 ± 0.01	0.5
$Cu-Fe_A$	4.0	0.008 ± 0.002	3.42 ± 0.02	1.0
$Cu-Fe_A$	2.0	0.009 ± 0.003	3.62 ± 0.03	1.0
Cu-O	2.0	0.040	3.557	1.0
Cu-O	4.0	0.040	3.633	1.0
Cu-O	2.0	0.040	3.705	1.0
$\mathbf{S_0^2}$ = 0.9 ± 0.1			$\mathbf{\Delta E_0}$ = -1.14 ± 1.26 eV	
		**R-factor** = 0.014		

* The distances involving the Cu^{2+} absorber with the ions located in the tetrahedral sites are split into two contributions with the same interatomic distances and Debye-Waller factors, as described in the main text. ** The distances involving the Cu^{2+} absorber with the ions located in the octahedral sites are split into two contributions with the same interatomic distances and Debye-Waller factors, as described in the main text.

Table S3 Best fit parameters obtained by fitting the experimental EXAFS of the XCuFe sample at the Cu K-edge with a 9 shell model of tetragonal copper ferrite. Coordination numbers (N), interatomic distances (R), Debye-Waller factors, S_0^2, ΔE_0 and R-factor are shown.

**XCuFe**			**Cu K-edge**	
**Abs.-Backscatter**	**N**	**σ^2(Å^2)**	**R(Å)**	**Occupancy**
Cu-O	4.0	0.006 ± 0.001	1.96 ± 0.01	1.0
Cu-O	2.0	0.011 ± 0.003	2.22 ± 0.02	1.0
$Cu-Cu_B$	2.0	0.006 ± 0.001	2.90 ± 0.02	0.5
$Cu-Cu_B$	4.0	0.011 ± 0.003	2.98 ± 0.02	0.5
$Cu-Fe_B$	2.0	0.006 ± 0.001	2.90 ± 0.02	0.5
$Cu-Fe_B$	4.0	0.011 ± 0.003	2.98 ± 0.02	0.5
$Cu-Fe_A$	4.0	0.010 ± 0.001	3.43 ± 0.02	1.0
$Cu-Fe_A$	2.0	0.011 ± 0.004	3.62 ± 0.04	1.0
Cu-O	2.0	0.040	3.557	1.0
Cu-O	4.0	0.040	3.633	1.0
Cu-O	2.0	0.040	3.705	1.0
$\mathbf{S_0^2}$ = 0.9			$\mathbf{\Delta E_0}$ = -0.49 ± 1.17 eV	
		**R-factor** = 0.014		

* The distances involving the Cu^{2+} absorber with the ions located in the tetrahedral sites are split into two contributions with the same interatomic distances and Debye-Waller factors, as described in the main text. ** The distances involving the Cu^{2+} absorber with the ions located in the octahedral sites are split into two contributions with the same interatomic distances and Debye-Waller factors, as described in the main text.

Table S4 Best fit parameters obtained by fitting the experimental EXAFS of the bulk-$CuFe_2O_4$ sample at the Fe K-edge with a 4 shell model for the tetrahedral site (A) and a 5 shell model for the octahedral site (B) using a cubic copper ferrite model. Coordination numbers (N), interatomic distances (R), Debye-Waller factors, S_0^2, ΔE_0 and R-factor are shown.

**Bulk $CuFe_2O_4$**		**Fe K-edge**		
**Abs. – Backscatter**	**N**	**σ^2(Å^2)**	**R(Å)**	**Occupancy**
Fe_A-O	4.0	0.005 ± 0.003	1.90 ± 0.05	0.50
Fe_A-Fe_B	12.0	0.009 ± 0.001	3.45 ± 0.02	0.25
Fe_A-Cu_B	12.0	0.009 ± 0.001	3.45 ± 0.02	0.25
Fe_A-O	12.0	0.02 ± 0.04	3.5 ± 0.2	0.50
Fe_A- Fe_A	4.0	0.005 ± 0.002	3.63 ± 0.02	0.50
Fe_B-O	6.0	0.011 ± 0.009	1.98 ± 0.04	0.50
Fe_B-Fe_B	6.0	0.007 ± 0.001	2.97 ± 0.01	0.25
Fe_B-Cu_B	6.0	0.007 ± 0.001	2.97 ± 0.01	0.25
Fe_B-Fe_A	6.0	0.009 ± 0.001	3.45 ± 0.02	0.50
Fe_B-O	2.0	0.02 ± 0.04	3.5 ± 0.2	0.50
Fe_B-O	6.0	0.04 ± 0.3	3.65	0.50
$\mathbf{S_0^2}$ = 0.7 ± 0.1			$\mathbf{\Delta E_0}$ = -2.86 ± 1.56 eV	
		**R-factor** = 0.011		

* The distances involving the Fe^{3+} absorber in the tetrahedral site with the ions located in the tetrahedral sites, and the distance involving the Fe^{3+} absorber in the octahedral site with the Cu^{2+} located in the tetrahedral sites, are split in contributions with the same interatomic distances and Debye-Waller factors, as described in the main text. ** The distances involving the Fe^{3+} absorber in the octahedral site with the ions located in the octahedral sites are split into two contributions, as described in the main text. *** The distances and Debye-Waller factors involving two Fe-O distances which are intrinsically different from a crystallographic point of view, but too close to be distinguished by EXAFS, were kept to the same values, as described in the main text.

Table S5 Best fit parameters obtained by fitting the experimental EXAFS of the XCuFe sample at the Fe K-edge with a 4 shell model for the tetrahedral site (A) and a 5 shell model for the octahedral site (B) using a cubic copper ferrite model. Coordination numbers (N), interatomic distances (R), Debye-Waller factors, S_0^2, ΔE_0 and R-factor are shown.

**XCuFe**		**Fe K-edge**		
**Abs. – Backscatter**	**N**	**σ^2(Å^2)**	**R(Å)**	**Occupancy**
Fe_A-O	4.0	0.001 ± 0.001	1.87 ± 0.01	0.50
Fe_A-Fe_B	12.0	0.008 ± 0.003	3.45 ± 0.04	0.25
Fe_A-Cu_B	12.0	0.008 ± 0.003	3.45 ± 0.04	0.25
Fe_A-O	12.0	0.01 ± 0.5	3.5 ± 0.1	0.50
Fe_A- Fe_A	4.0	0.005 ± 0.004	3.59 ± 0.07	0.50
Fe_B-O	6.0	0.003 ± 0.001	1.99 ± 0.01	0.50
Fe_B-Fe_B	6.0	0.007 ± 0.001	2.97 ± 0.01	0.25
Fe_B-Cu_B	6.0	0.007 ± 0.001	2.97 ± 0.01	0.25
Fe_B-Fe_A	6.0	0.008 ± 0.003	3.45 ± 0.04	0.50
Fe_B-O	2.0	0.01 ± 3	3.5 ± 0.1	0.50
Fe_B-O	6.0	0.03 ± 0.3	3.65	0.50
$\mathbf{S_0^2}$ = 0.7			$\mathbf{\Delta E_0}$ = -2.96 ± 0.80 eV	
		**R-factor** = 0.011		

* The distances involving the Fe^{3+} absorber in the tetrahedral site with the ions located in the tetrahedral sites, and the distance involving the Fe^{3+} absorber in the octahedral site with the Cu^{2+} located in the tetrahedral sites, are split in contributions with the same interatomic distances and Debye-Waller factors, as described in the main text. ** The distances involving the Fe^{3+} absorber in the octahedral site with the ions located in the octahedral sites are split into two contributions, as described in the main text. *** The distances and Debye-Waller factors involving two Fe-O distances which are intrinsically different from a crystallographic point of

view, but too close to be distinguished by EXAFS, were kept to the same values, as described in the main text.

References

S1. Brunauer, S., Emmett, P. H. & Teller, E. Adsorption of gases in multimolecular layers. *J. Am. Chem. Soc.* **60**(2), 309-319 (1938).

S2. Barrett, E. P., Joyner, L. G. & Halenda, P. P. The determination of pore volume and area distributions in porous substances. I. Computations from nitrogen isotherms. *J. Am. Chem. Soc.* **73**(1), 373-380 (1951).

S3. Rouquerol, F., Rouquerol, J. & Sing, K. Adsorption by powders and porous solids: principles, methodology and applications (ed. Academic Press) London (1999).
